# Incongruous consultation behaviour: results from a UK-wide population survey

**DOI:** 10.1186/1471-2296-13-21

**Published:** 2012-03-20

**Authors:** Alison M Elliott, Anne McAteer, Philip C Hannaford

**Affiliations:** 1 Centre of Academic Primary Care Division of Applied Health Sciences, University of Aberdeen, Polwarth Building, West Block Foresterhill, Aberdeen AB25 2ZD, UK

**Keywords:** Signs & symptoms, Community-based, Health care services, Primary care

## Abstract

**Background:**

Symptom characteristics are strong drivers of care seeking. Despite this, incongruous consultation behaviour occurs and has implications for both individuals and health-care services. The aim of this study was to determine how frequently incongruous consultation behaviour occurs, to examine whether it is more common for certain types of symptoms and to identify the factors associated with being an incongruous consulter.

**Methods:**

An age and sex stratified random sample of 8,000 adults was drawn from twenty UK general practices. A postal questionnaire was used to collect detailed information on the presence and characteristics of 25 physical and psychological symptoms, actions taken to manage the symptoms, general health, attitudes to symptom management and demographic/socio-economic details. Two types of incongruous consultation behaviour were examined: i) consultation with a GP for symptoms self-rated as low impact and ii) no consultation with a GP for symptoms self-rated as high impact.

**Results:**

A fifth of all symptoms experienced resulted in consultation behaviour which was incongruous based on respondents' own rating of the symptoms' impact. Low impact consultations were not common, although symptoms indicative of a potentially serious condition resulted in a higher proportion of low impact consultations. High impact non-consultations were more common, although there was no clear pattern in the type of associated symptoms. Just under half of those experiencing symptoms in the previous two weeks were categorised as an incongruous consulter (low impact consulter: 8.3%, high impact non-consulter: 37.1%). Employment status, having a chronic condition, poor health, and feeling that reassurance or advice from a health professional is important were associated with being a low impact consulter. Younger age, employment status, being an ex-smoker, poor health and feeling that not wasting the GPs time is important were associated with being a high impact non-consulter.

**Conclusions:**

This is one of the first studies to examine incongruous consultation behaviour for a range of symptoms. High impact non-consultations were common and may have important health implications, particularly for symptoms indicative of serious disease. More research is now needed to examine incongruous consultation behaviour and its impact on both the public's health and health service use.

## Background

Symptoms are both common and powerful drivers of healthcare utilisation [1,2]. Presentation of symptoms to general practice is patient expressed need and is often a marker of ill health. While the decision to consult a general practitioner (GP) results from a complex mix of physical, psychological and social factors [3], previous research suggests that symptom characteristics (including severity and interference with daily life) are the strongest drivers of care seeking; being more consistently related to seeking help than patient characteristics such as demographic and socio-economic factors [4-10]. Despite this, incongruous consultation behaviour occurs in general practice and has important implications for both individuals and health-care services.

Two examples of incongruous consultation behaviours are: i) consultation with a GP for symptoms self-rated as low impact (designated here as "low impact consultations") and ii) no consultation with a GP for symptoms self-rated as high impact (designated here as "high impact non-consultations"). Individuals in the high impact non-consultation group may have potentially serious symptoms which, for whatever reason, are not presented to a GP. Understanding this group is important for several reasons. Firstly, some individuals with known medical problems may have poor symptom control which impacts on quality of life and other health outcomes. Secondly, some individuals may have unknown medical conditions which are inevitably left untreated, with potentially serious consequences [11,12]. While there may be less concern regarding the immediate health implications for individuals in the low impact consultation group, this group is still important. Consultations involving only low impact symptoms may increase an individual's concern about such symptoms and inadvertently reinforce them as important. In addition, low impact consultations may place an unnecessary burden on busy healthcare systems, possibly resulting in delays to services for those with symptoms suggestive of more serious disease.

There has been comparatively little research to date examining incongruous consultation behaviour. One possible reason for this is the difficulty in defining the concept. In this paper we examine consultation behaviour which is incongruous based on respondents' own rating of the symptoms' impact. It is important to note that the term 'incongruous' and not the term 'inappropriate' is used to refer to the consultation behaviour. Without important contextual and longitudinal information it is often impossible to judge whether a consultation is appropriate or not. Furthermore, opinions on appropriateness differ widely depending on whether an individual patient, family, healthcare professional, societal, or policy perspective is taken. This paper determines how frequently incongruous consultation behaviour occurs in UK general practice, examines whether it is more common for certain types of symptoms and identifies factors associated with being an incongruous consulter. We were particularly interested in identifying factors which may explain why some people seek help for low impact symptoms and why some people do not seek help for high impact symptoms.

## Methods

### Subjects and sampling

**Figure 1:**
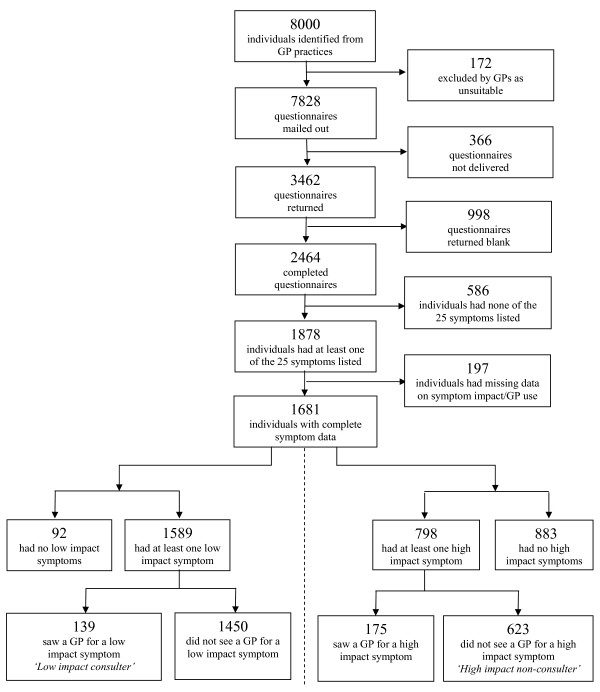
**Flow chart of study sample**. NB. Since individuals could have both low and high impact symptoms the dotted vertical line represents a split in the flowchart. The left-hand side shows the 1681 individuals divided into those without and with low impact symptoms and whether the latter consulted about them or not. The right-hand side shows the same information about high impact symptoms. Of the 1681 individuals with symptoms, 244 consulted a GP about at least one symptom. Of these, 39 individuals were both a low impact consulter and a high impact non-consulter and 59 individuals were both a low impact consulter and a high impact consulter.

A UK-wide population-based postal survey was undertaken in 2007/2008. Full details of the sample and methods have been published previously [13]. In brief, an age- and sex-stratified random sample of 8,000 adults aged 18-60 years was drawn from 20 general practices across the UK following confirmation of ethical approval (Figure 1). Practices were recruited from the nationally representative Medical Research Council General Practice Research Framework and varied in their size, geographical location, area type and level of deprivation. GPs screened the sample and excluded anyone whom they felt it would be insensitive or inappropriate to approach. Practice research nurses sent out questionnaire packs and covering letters on our behalf. A reminder letter and replacement questionnaire was sent to non-respondents after three weeks.

### Questionnaire

The questionnaire enquired about 25 physical and psychological symptoms experienced in the previous two weeks. Symptoms were identified from previous literature and pilot work, and ranged in seriousness (based on ratings by a sample of local GPs) from level 1: symptoms usually indicative of minor or self-limiting illness (e.g. sore throat, feeling tired/run down, diarrhoea) through to level 5: symptoms which could be indicative of potentially serious conditions (i.e. chest pain and coughing up blood) [4]. A two week time period was examined as this was considered long enough to enable many of the symptoms to have lasted their full duration and for actions to have been taken, but short enough to ensure good recall of symptom occurrence and associated responses. For each symptom experienced in the previous two weeks, respondents were asked about the severity of the symptom at its worst (using a standard 5 point severity scale); how much it had interfered with daily life (using a standard 5 point interference scale); and whether or not they had consulted a general practitioner about the symptom in the last two weeks. Comprehensive data were also collected on demographic and socio-economic factors including sex, age, marital status, social support, education, housing, employment, household income, ethnicity, smoking, access to a GP surgery, access to a pharmacy and access to a shop selling over-the-counter (OTC) medicines.

Health factors measured included the presence of a chronic condition and general health. The presence of a chronic condition was ascertained by asking participants if they currently had any of 14 conditions: asthma; chronic bronchitis; other chest trouble; diabetes; cancer; stroke; epilepsy or fits; stomach/digestive disorder; depression/nervous trouble; other mental health problems; high blood pressure; heart troubles; liver troubles and rheumatic trouble/arthritis. Individuals responding positively to any one of these conditions were categorised as having a current chronic condition. General health was measured using the Short Form 36 (SF-36) [14,15] questionnaire. The SF-36 is a well validated and widely used instrument which measures an individual's perception of their health across eight separate health domains, with higher scores representing better health.

Symptom management factors were measured using the Nijmegen Expectation Questionnaire (NEQ) and a series of management statements. The NEQ is a previously validated questionnaire comprising 12 questions designed to ascertain individuals' attitudes towards the management of common symptoms, in particular whether they are better treated by consulting a GP as compared to self-care [16]. Higher scores represent stronger beliefs about the benefits of treatment by the GP compared to self-care. A list of 15 management statements were also developed to identify people's views on what influences their symptom management and included personal factors, social factors, factors relating to the symptom and factors relating to healthcare services. Participants were asked to indicate whether they believed these factors were important or not important when deciding how to manage their symptoms.

### Analysis

Data were analysed in two different ways: a) at the symptom level and b) at the person level. At the symptom level, descriptive statistics were used to examine the proportion of symptoms resulting in incongruous consultation behaviour over the previous two weeks. Low impact consultations were those associated with symptoms self-rated by respondents as low severity (1-3 on the standard 5-point severity scale) and causing no or little interference (1-3 on the standard 5-point interference scale) and which resulted in a GP consultation. High impact non-consultations were those associated with symptoms self-rated by respondents as high severity (4 or 5 on the standard 5-point severity scale) and/or causing considerable interference (4 or 5 on the standard 5-point interference scale) which did not result in a GP consultation. Incongruous consultation behaviour was then examined by type of symptom to determine whether it is more common among certain types of symptoms.

Subsequent analysis examined data at the person level. Individuals were classified as a "low impact consulter" if they had at least one low impact symptom in the previous two weeks which they consulted their GP about. Individuals were classified as a "high impact non-consulter" if they had at least one high impact symptom in the previous two weeks which they did not consult their GP about. Descriptive statistics examined the proportion of people categorised as low impact consulters and high impact non-consulters and the number of symptoms resulting in incongruous consultation behaviour. Binary logistic regression was used to examine the factors associated with being a low impact consulter and the factors associated with being a high impact non-consulter. Age and sex adjusted odds ratios, and their associated 95% confidence intervals, were calculated for each factor of interest. Backward stepwise logistic regression modelling was then used to build a multi-variable model to identify which factors were independently associated with each of the two incongruous consultation behaviour groups. Factors were included in the multivariate modelling if they had a significant association (p < 0.05) with the outcome of interest after adjustment for age and sex. Since individuals could be categorised as a low impact consulter for one symptom and a high impact non-consulter for another symptom, low impact consulters could not be directly compared with high impact non-consulters as the two groups were not mutually exclusive. To ensure that the groups being compared comprised independent observations those identified as a low impact consulter in the previous two weeks were compared against all those with symptoms in the last two weeks who were not categorised as a low impact consulter. Similarly, those identified as a high impact non-consulter in the previous two weeks were compared against all those with symptoms in the previous two weeks who were not categorised as a high impact non-consulter.

## Results

### Response rate and sample characteristics

Full details of the response rate, sample characteristics and symptom prevalence have been published previously [13]. A total of 46.4% of questionnaires were returned, of which 2,474 were completed, giving a corrected completed response rate of 33.2% (Figure 1). Most demographic and socio-economic groups (except non-whites) were well represented in the sample [13]. Full details of the patterns of symptoms reported and patterns of consultation behaviour in the sample have also been published previously [4,13]. In brief, just over three-quarters of the sample reported experiencing symptoms in the last two weeks. Those in the younger age groups, those with a chronic condition and those unable to work due to illness or not in paid work were most likely to report symptoms. A total of 8% of all symptoms experienced resulted in consultation with a GP in the last two weeks. Individuals no longer married, those unable to work due to illness, those with a chronic condition and those with a higher number of symptoms were most likely to have consulted a GP about their symptoms.

### Incongruous consultation behaviour (symptom level analysis)

A fifth (n = 1638) of the 7995 symptoms experienced in the previous two weeks resulted in incongruous consultation behaviour based on respondents' own rating of the symptoms' impact (Table 1). A total of 254 (3.2%) symptoms resulted in a low impact consultation. Although many of the low impact consultations occurred for symptoms indicative of long term chronic conditions (such as *joint pain *and *back pain*) or minor, self-limiting symptoms (such as *sore throat, cough *and *indigestion/heartburn*) it was symptoms which could be indicative of a potentially serious condition (such as *blood in stool, fainting, wheezy chest, shortness of breath, unintentional weight loss *and *chest pain*) that had the largest proportion of low impact consultations.

**Table 1:** Low impact consultations and high impact non-consultations in the previous two weeks by symptom

Level of symptom seriousness *	Symptom	n	**Low impact consultations **†		**High impact non-consultations **††	
			
			n	%	n	%
1	Feeling tired/run down	887	12	1.4	201	22.7
	Difficulty sleeping	607	12	2.0	155	25.5
	Sore throat	411	17	4.1	39	9.5
	Cold or flu symptoms	372	9	2.4	52	14.0
	Diarrhoea	266	9	3.4	37	13.9
	Loss of appetite	117	6	5.1	16	13.7

2	Back pain	653	15	2.3	144	22.1
	Nervousness/anxiety	405	10	2.5	88	21.7
	Cough	372	12	3.2	48	12.9
	Nausea/feeling sick	259	6	2.3	36	13.9
	Constipation	203	4	2.0	11	5.4
	Vomiting	95	2	2.1	24	25.3

3	Headaches	845	5	0.6	143	16.9
	Joint pain	678	30	4.4	141	20.8
	Indigestion/heartburn	392	13	3.3	41	10.5
	Feeling depressed	353	12	3.4	100	28.3
	Stomach/abdominal pain	337	20	5.9	66	19.6
	Dizziness	194	9	4.6	21	10.8
	Wheezy chest	158	14	8.9	16	10.1
	Fainting	14	2	14.3	4	28.6

4	Shortness of breath	176	15	8.5	23	13.1
	Blood in stool	52	9	17.3	1	1.9
	Unintentional weight loss	37	3	8.1	5	13.5

5	Chest pain	108	8	7.4	12	11.1
	Coughing up blood	4	0	0	1	25.0

**All symptoms combined**		**7995**	**254**	**3.2**	**1384**	**17.3**

* Level of symptom seriousness perceived by a sample of GPs, where level 1 is least serious and includes symptoms usually indicative of trivial/self-limiting illness and level 5 is most serious and includes symptoms that could be indicative of a serious condition or illness

† Consulted GP for symptoms self-rated as low severity (1-3 on a standard 5-point severity scale) ***and ***causing no or little interference on daily life (1-3 on a standard 5-point interference scale)

†† Did not consult GP for symptoms self-rated as high severity (4 or 5 on a standard 5 point severity scale) ***and/or ***causing considerable interference on daily life (4 or 5 on a standard 5-point interference scale)

A total of 1384 (17.3%) symptoms resulted in a high impact non-consultation. There was no clear pattern in the symptoms resulting in a high impact non-consultation, with *fainting, feeling depressed, difficulty sleeping, vomiting *and *coughing up blood *the symptoms most commonly resulting in a high impact non-consultation. For each of the individual symptoms investigated (with the exception of *blood in stool*) the proportion of high impact non-consultations was larger than the proportion of low impact consultations.

### Incongruous consultation behaviour groups (person level analysis)

Of the 1681 respondents who experienced at least one symptom in the previous two weeks and who provided full information on symptom impact and GP use, just under half (45.4%) were categorised into one of the two incongruous consultation behaviour groups (Figure 1). A total of 139 individuals (8.3%) reported at least one low impact symptom in the previous two weeks which they consulted their GP about and so were categorised as low impact consulters. Most individuals were a low impact consulter for one (61.2%) or two (21.6%) symptoms. The maximum number of symptoms that an individual was a low impact consulter for was 10. A total of 623 individuals (37.1%) reported at least one high impact symptom in the previous two weeks which they did not consult their GP about and so were categorised as high impact non-consulters. Most of these were a high impact non-consulter for one (43.7%) or two (25.2%) symptoms. The maximum number of symptoms that an individual was a high impact non-consulter for was 15. Few individuals (37, 2.2%) were categorised as both a low impact consulter for one symptom and a high impact non-consulter for another symptom.

### Factors associated with being a low impact consulter

Few demographic and socio-economic factors were significantly associated with being a low impact consulter after adjustment for age and sex (Table 2). Individuals with higher education qualifications and those who were self-employed were significantly less likely to be a low impact consulter than those with no educational qualifications and those working full time respectively. Individuals with poor access to a pharmacy and those with poor access to a shop selling OTC medicines were significantly more likely to be a low impact consulter than those with good access to these services.

**Table 2:** Associations between low impact consulters and high impact non-consulters and demographic/socio-economic factors (age and sex adjusted odds ratios)

Demographic and socio-economic factors	Low impact consulters		High impact non-consulters	
	
	Odds ratio (95% CI)	p- value	Odds ratio (95% CI)	p-value
**Sex**				
Males^†^	1.00		1.00	
Females	1.26 (0.87 to 1.83)	0.220	1.20 (0.97 to 1.48)	0.087

**Age**				
18-24 yrs^†^	1.00		1.00	
25-34 yrs	0.81 (0.40 to 1.65)	0.556	0.93 (0.62 to 1.40)	0.731
35-44 yrs	0.71 (0.37 to 1.38)	0.308	0.59 (0.40 to 0.86)	0.006
45-54 yrs	0.88 (0.46 to 1.68)	0.699	0.63 (0.43 to 0.92)	0.016
55-60 yrs	1.14 (0.60 to 2.19)	0.684	0.67 (0.45 to 0.99)	0.043

**Marital status**				
Single^†^	1.00		1.00	
Married/living together	0.83 (0.49 to 1.41)	0.490	0.87 (0.64 to 1.17)	0.354
No longer married	1.13 (0.54 to 2.33)	0.750	1.75 (1.13 to 2.71)	0.012

**Social support**				
Low^†^	1.00		1.00	
Medium	0.94 (0.43 to 2.07)	0.876	0.51 (0.32 to 0.79)	0.003
High	0.87 (0.40 to 1.88)	0.717	0.44 (0.28 to 0.69)	< 0.001

**Education**				
No qualifications^†^	1.00		1.00	
Secondary school	0.82 (0.48 to 1.41)	0.480	0.56 (0.40 to 0.79)	0.001
Higher education	0.54 (0.31 to 0.95)	0.033	0.42 (0.30 to 0.60)	< 0.001

**Housing**				
Owned/mortgaged^†^	1.00		1.00	
Privately rented and other	1.02 (0.57 to 1.85)	0.938	1.29 (0.93 to 1.80)	0.131
Council/housing association	1.14 (0.59 to 2.19)	0.693	2.80 (1.91 to 4.11)	< 0.001

**Employment**				
Full-time^†^	1.00		1.00	
Part-time	0.82 (0.50 to 1.35)	0.436	1.09 (0.81 to 1.46)	0.572
Self-employed	0.31 (0.11 to 0.86)	0.024	1.53 (1.05 to 2.24)	0.029
Not working due to illness	1.44 (0.74 to 2.81)	0.289	9.19 (5.32 to 15.87)	< 0.001
Others not in employment	0.67 (0.39 to 1.15)	0.146	1.42 (1.06 to 1.91)	0.021

**Household income (annual)**				
< £15,000^†^	1.00		1.00	
£15,000-29,999	0.89 (0.50 to 1.56)	0.672	0.77 (0.55 to 1.08)	0.135
£30,000-49,000	0.81 (0.46 to 1.42)	0.459	0.48 (0.34 to 0.67)	< 0.001
£50,000+	0.71 (0.40 to 1.28)	0.256	0.33 (0.23 to 0.47)	< 0.001

**Ethnicity**				
White^†^	1.00		1.00	
Other	0.54 (0.13 to 2.25)	0.393	0.99 (0.53 to 1.83)	0.966

**Smoking**				
Never^†^	1.00		1.00	
Ex-smoker	1.00 (0.65 to 1.55)	0.991	1.70 (1.33 to 2.17)	< 0.001
Current smoker	1.14 (0.72 to 1.82)	0.579	2.00 (1.53 to 2.60)	< 0.001

**Access to a GP surgery**				
Good^†^	1.00		1.00	
Moderate	0.98 (0.64 to1.50)	0.938	1.32 (1.04 to 1.68)	0.021
Poor	1.04 (0.40 to 2.66)	0.942	1.74 (1.02 to 2.96)	0.042

**Access to a pharmacy**				
Good^†^	1.00		1.00	
Moderate	1.34 (0.85 to 2.11)	0.210	1.24 (0.94 to 1.63)	0.125
Poor	2.74 (1.17 to 6.38)	0.020	1.26 (0.66 to 2.42)	0.482

**Access to a shop selling** **OTC medicines**				
Good^†^	1.00		1.00	
Moderate	1.17 (0.71 to 1.93)	0.544	1.16 (0.86 to 1.55)	0.335
Poor	3.26 (1.28 to 8.33)	0.013	1.91 (0.89 to 4.11)	0.100

† Referent group

Figures highlighted are significant at 5% level (p < 0.05)

All of the health factors examined were significantly associated with being a low impact consulter (Table 3). After adjustment for age and sex, individuals with a chronic condition were more than twice as likely to be a low impact consulter than those without an existing chronic condition. Individuals with the poorest health scores in all eight of the SF-36 domains were significantly more likely to be a low impact consulter than those with the best health scores.

**Table 3:** Associations between low impact consulters and high impact non-consulters and health factors (age and sex adjusted odds ratios)

Health factors	Low impact consulters		High impact non-consulters	
	
	Odds ratio (95% CI)	p-value	Odds ratio (95% CI)	p-value
**Chronic condition**				
No^†^	1.00		1.00	
Yes	2.37 (1.61 to 3.50)	< 0.001	2.23 (1.80 to 2.76)	< 0.001

**Physical functioning**				
Quartile 1 (best health)^†^	1.00		1.00	
Quartile 2	1.83 (1.07 to 3.11)	0.027	1.19 (0.87 to 1.63)	0.273
Quartile 3	1.55 (0.91 to 2.65)	0.108	1.57 (1.17 to 2.11)	0.003
Quartile 4 (poorest health)	2.39 (1.49 to 3.85)	< 0.001	3.49 (4.23 to 3.20)	< 0.001

**Role physical**				
Quartile 1 (best health)^†^	1.00		1.00	
Quartile 2	1.56 (0.82 to 2.99)	0.175	1.16 (0.79 to 1.69)	0.460
Quartile 3	1.40 (0.83 to 2.37)	0.206	2.32 (1.76 to 3.07)	< 0.001
Quartile 4 (poorest health)	3.28 (2.11 to 5.09)	< 0.001	6.67 (5.06 to 8.78)	< 0.001

**Bodily pain**				
Quartile 1 (best health)^†^	1.00		1.00	
Quartile 2	1.24 (0.51 to 2.98)	0.638	2.09 (1.37 to 3.17)	0.001
Quartile 3	3.37 (1.97 to 5.76)	< 0.001	2.96 (2.19 to 4.01)	< 0.001
Quartile 4 (poorest health)	3.63 (2.14 to 6.17)	< 0.001	11.10 (8.17 to 15.07)	< 0.001

**General health**				
Quartile 1 (best health)^†^	1.00		1.00	
Quartile 2	1.61 (0.90 to 2.90)	0.109	1.19 (0.87 to 1.63)	0.279
Quartile 3	1.66 (0.86 to 3.21)	0.134	1.76 (1.24 to 2.51)	0.002
Quartile 4 (poorest health)	2.71 (1.57 to 4.66)	< 0.001	4.27 (3.18 to 5.74)	< 0.001

**Vitality**				
Quartile 1 (best health)^†^	1.00		1.00	
Quartile 2	0.96 (0.51 to 1.82)	0.902	1.44 (1.01 to 2.04)	0.042
Quartile 3	1.63 (0.94 to 2.85)	0.085	2.28 (1.65 to 3.15)	< 0.001
Quartile 4 (poorest health)	2.68 (1.55 to 4.64)	< 0.001	7.12 (5.08 to 9.98)	< 0.001

**Social functioning**				
Quartile 1 (best health)^†^	1.00		1.00	
Quartile 2	1.44 (0.76 to 2.72)	0.264	1.33 (0.91 to 1.95)	0.139
Quartile 3	1.75 (1.06 to 2.90)	0.030	2.82 (2.11 to 3.77)	< 0.001
Quartile 4 (poorest health)	2.55 (1.65 to 3.94)	< 0.001	7.93 (6.03 to 10.43)	< 0.001

**Role emotional**				
Quartile 1 (best health)^†^	1.00		1.00	
Quartile 2	1.12 (0.77 to 1.61)	0.487	1.09 (0.79 to 1.42)	0.635
Quartile 3	1.48 (0.90 to2.43)	0.118	1.26 (0.93 to1.70)	0.133
Quartile 4 (poorest health)	1.69 (1.14 to 2.50)	0.009	3.17 (2.51 to 4.00)	< 0.001

**Mental health**				
Quartile 1 (best health)^†^	1.00		1.00	
Quartile 2	1.25 (0.76 to 2.05)	0.386	1.00 (0.74 to 1.36)	0.992
Quartile 3	0.91 (0.50 to 1.66)	0.764	2.05 (1.49 to 2.81)	< 0.001
Quartile 4 (poorest health)	1.72 (1.09 to2.73)	0.021	3.93 (2.97 to 5.20)	< 0.001

† Referent group

Figures highlighted are significant at 5% level (p < 0.05)

Few of the symptom management factors examined were significantly associated with being a low impact consulter (Table 4). After adjustment for age and sex, individuals with the highest NEQ scores (representing a belief that GP treatment is better than self-care for common symptoms) were more than twice as likely to be a low impact consulter as those with the lowest NEQ scores. Individuals who believed that reassurance/advice from a health professional was important when deciding how to manage symptoms were also more than twice as likely to be a low impact consulter as those who felt this was not important.

**Table 4:** Associations between low impact consulters and high impact non-consulters and views on managing symptoms (age and sex adjusted odds ratios)

Symptom management factors	Low impact consulters		High impact non-consulters	
	
	Odds ratio (95% CI)	p-value	Odds ratio (95% CI)	p-value
**Nijmegen Expectation** **Questionnaire ^**				
Quartile 1 (lowest scores)^†^	1.00		1.00	
Quartile 2	1.75 (0.97 to 3.16)	0.062	1.24 (0.93 to 1.66)	0.139
Quartile 3	2.29 (1.32 to 3.98)	0.003	1.24 (0.93 to 1.64)	0.142
Quartile 4 (highest scores)	2.78 (1.59 to 4.86)	< 0.001	1.51 (1.13 to 2.02)	0.006

**My previous experience** **of a symptom**				
Not important^†^	1.00		1.00	
Important	1.02 (0.36 to 2.89)	0.969	0.94 (0.53 to 1.68)	0.845

**Other people's experiences** **of a symptom**				
Not important^†^	1.00		1.00	
Important	1.08 (0.75 to 1.54)	0.684	1.14 (0.93 to 1.40)	0.214

**My knowledge/beliefs about** **a symptom**				
Not important^†^	1.00		1.00	
Important	0.53 (0.27 to1.08)	0.079	0.97 (0.59 to1.60)	0.916

**Feeling a symptom wouldn't be** **viewed as important by others**				
Not important^†^	1.00		1.00	
Important	1.18 (0.83 to 1.69)	0.350	1.34 (1.09 to 1.64)	0.005

**Feeling able to deal with** **a symptom myself**				
Not important^†^	1.00		1.00	
Important	0.65 (0.29 to 1.47)	0.304	0.96 (0.56 to 1.63)	0.872

**Feeling that I need reassurance or** **advice from a health professional**				
Not important^†^	1.00		1.00	
Important	2.61 (1.39 to 4.91)	0.003	1.09 (0.84 to 1.42)	0.506

**Feeling that I don't want to** **waste the GP's time**				
Not important^†^	1.00		1.00	
Important	0.99 (0.66 to 1.50)	0.976	1.60 (1.25 to 2.05)	< 0.001

**My knowledge about healthcare** **services and medicines**				
Not important^†^	1.00		1.00	
Important	1.53 (0.89 to 2.64)	0.126	1.01 (0.77 to 1.32)	0.963

**My attitudes/beliefs about** **healthcare services and medicines**				
Not important^†^	1.00		1.00	
Important	0.93 (0.62 to 1.38)	0.705	1.11 (0.88 to 1.40)	0.373

**My previous experience of** **healthcare services and medicines**				
Not important^†^	1.00		1.00	
Important	1.21 (0.76 to 1.92)	0.429	1.35 (1.05 to 175)	0.020

**Other people's experiences of** **healthcare services and medicines**				
Not important^†^	1.00		1.00	
Important	0.96 (0.67 to 1.37)	0.815	1.37 (1.12 to 1.68)	0.002

**The cost of using a particular** **healthcare service or treatment**				
Not important^†^	1.00		1.00	
Important	1.11 (0.78 to 1.57)	0.576	1.16 (0.95 to 1.42)	0.140

**The length of time I have** **to wait to get treatment**				
Not important^†^	1.00		1.00	
Important	1.03 (0.68 to 1.55)	0.898	1.24 (0.98 to 1.58)	0.076

**Availability of resources like** **transport and childcare**				
Not important^†^	1.00		1.00	
Important	0.87 (0.61 to 1.24)	0.449	1.52 (1.24 to 1.86)	< 0.001

**Information or advice** **appearing in the media**				
Not important^†^	1.00		1.00	
Important	1.08 (0.76 to 1.53)	0.671	0.99 (0.81 to 1.21)	0.922

† Referent group

Figures highlighted are significant at 5% level (p < 0.05)

**^ **Where lower scores represent a belief that self-care is better than GP treatment for common symptoms and higher scores represent a belief that GP treatment is better than self-care for common symptoms

Table 5 presents the final multivariate model of factors significantly and independently associated with being a low impact consulter. Individuals with an existing chronic condition, those with the poorest role physical scores, those with poor bodily pain scores, and those believing that reassurance or advice from a health professional is important were all more likely to be a low impact consulter than those in the reference group of each of these factors, while those who were self-employed were less likely to be a low impact consulter than those employed full-time.

**Table 5:** Multivariate model of factors significantly associated with being a low impact consulter

	Odds ratio (95% CI)	p-value
**Employment**		
Full-time^†^	1.00	
Part-time	0.77 (0.46 to 1.29)	0.324
Self-employed	0.29 (0.10 to 0.82)	0.019
Not working due to illness	0.62 (0.28 to 1.36)	0.230
Others not in employment	0.59 (0.34 to 1.04)	0.067

**Chronic condition**		
No^†^	1.00	
Yes	2.00 (1.32 to 3.04)	0.001

**Role physical**		
Quartile 1 (best health)^†^	1.00	
Quartile 2	1.16 (0.58 to 2.34)	0.678
Quartile 3	1.12 (0.64 to 1.96)	0.691
Quartile 4 (poorest health)	2.27 (1.32 to 3.90)	0.003

**Bodily pain**		
Quartile 1 (best health)^†^	1.00	
Quartile 2	0.81 (0.29 to 2.24)	0.681
Quartile 3	2.86 (1.63 to 5.03)	< 0.001
Quartile 4 (poorest health)	2.00 (1.07 to 3.73)	0.030

**Feeling that I need reassurance or advice from a health professional**		
Not important^†^	1.00	
Important	2.60 (1.33 to 5.08)	0.005

† Referent group

Figures highlighted are significant at 5% level (p < 0.05)

### Factors associated with being a high impact non-consulter

Most of the demographic and socio-economic factors examined were significantly associated with being a high impact non-consulter after adjustment for age and sex (Table 2). Older age groups, those with medium or high social support, those with secondary school or higher education qualifications and those with an annual household income of £30,000 or more were significantly less likely to be a high impact non-consulter than those in the reference group of each of these factors. Those no longer married, those living in council/housing association accommodation, those self-employed or unable to work due to illness, current and ex smokers and those with poor or moderate access to a GP surgery were significantly more likely to be a high impact non-consulter than those in the reference group of each of these factors.

After adjustment for age and sex, all the health factors examined were significantly associated with being a high impact non-consulter (Table 3). Individuals with a chronic condition were more than twice as likely to be a high impact non-consulter as those without an existing chronic condition. Individuals with the poorest health scores in all eight of the SF-36 health domains were significantly more likely to be a high impact non-consulter than those with the highest health scores.

A number of the symptom management factors were significantly associated with being a high impact non-consulter (Table 4). After adjustment for age and sex, individuals with the highest NEQ scores (representing a belief that GP treatment is better than self-care for common symptoms) were significantly more likely to be a high impact non-consulter than those with the lowest NEQ scores. Those feeling that a symptom would not be viewed as important by others, those feeling that it is important not to waste the GP's time, those who believe their previous experience of healthcare is important, those who believe other people's experiences of healthcare is important and those who say that the availability of resources such as transport and childcare is important when deciding how to manage symptoms were all significantly more likely to be a high impact non-consulter than those who felt these issues were not important.

Table 6 presents the final multivariate model of factors independently associated with being a high impact non-consulter. Individuals not working due to illness, ex-smokers, those with poor bodily pain scores, those with the poorest vitality scores, those with poor social functioning scores and those who believe it is important not to waste the GP's time were all more likely to be a high impact non-consulter than those in the reference group for each of these characteristics. Older participants were less likely to be high impact non-consulters than younger participants.

**Table 6:** Multivariate model of factors significantly associated with being a high impact non-consulter

	Odds ratio (95% CI)	p-value
**Age**		
18-24 yrs^†^	1.00	
25-34 yrs	1.17 (0.69 to 1.99)	0.571
35-44 yrs	0.53 (0.32 to 0.87)	0.013
45-54 yrs	0.51 (0.31 to 0.85)	0.009
55-60 yrs	0.49 (0.29 to 0.82)	0.007

**Employment**		
Full-time^†^	1.00	
Part-time	1.03 (0.72 to 1.46)	0.889
Self-employed	1.33 (0.81 to 2.16)	0.261
Not working due to illness	2.89 (1.27 to 6.56)	0.011
Others not in employment	1.20 (0.82 to 1.74)	0.348

**Smoking**		
Never^†^	1.00	
Ex-smoker	1.52 (1.11 to 2.08)	0.010
Current smoker	1.22 (0.86 to 1.73)	0.258

**Bodily pain**		
Quartile 1 (best health)^†^	1.00	
Quartile 2	1.62 (0.99 to 2.64)	0.053
Quartile 3	2.54 (1.79 to 3.59)	< 0.001
Quartile 4 (poorest health)	5.96 (4.12 to 8.62)	< 0.001

**Vitality**		
Quartile 1 (best health)^†^	1.00	
Quartile 2	0.98 (0.65 to 1.50)	0.941
Quartile 3	1.07 (0.71 to 1.61)	0.753
Quartile 4 (poorest health)	2.09 (1.33 to 3.31)	0.002

**Social functioning**		
Quartile 1 (best health)^†^	1.00	
Quartile 2	1.01 (0.64 to 1.57)	0.983
Quartile 3	1.97 (1.38 to 2.81)	< 0.001
Quartile 4 (poorest health)	2.65 (1.80 to 3.90)	< 0.001

**Feeling that I don't want to waste the GP's time**		
Not important^†^	1.00	
Important	1.58 (1.15 to 2.16)	0.004

† Referent group

Figures highlighted are significant at 5% level (p < 0.05)

## Discussion

### Summary of main findings

This study has shown that around a fifth of all symptoms occurring in the community result in consultation behaviour which is incongruous based on respondents' own rating of their symptoms' impact. Low impact consultations were not common, although symptoms indicative of a potentially serious condition resulted in a higher proportion of low impact consultations. High impact non-consultations were more common, but there was no clear pattern in the types of associated symptoms. Just under half of those experiencing symptoms in the previous two weeks were categorised into one of the two incongruous consultation behaviour groups, mostly high impact non-consulters. Employment status, having a chronic condition, poor health, and feeling that reassurance or advice from a health professional is important were the factors associated with being a low impact consulter. Younger age, employment status, being an ex-smoker, poor health and feeling that not wasting the GPs time is important were the factors associated with being a high impact non-consulter.

### Strengths and limitations of the study

This is one of the first studies to examine incongruous consultation behaviour for a range of symptoms. The study provides a clear objective definition of incongruous consultation behaviour, which up till now has been lacking in the primary care symptom research literature. This objective definition will be useful for future research and will allow direct comparisons between studies. Our study provides important information about the frequency of incongruous consultations, what types of symptoms result in incongruous consultations, and what factors are associated with being a low impact consulter and a high impact non-consulter. The study included a wide range of symptoms, including physical and psychological symptoms, with symptoms ranging from those usually indicative of minor illness to those which could be indicative of a serious condition.

In this study, the respondents' own rating of the perceived severity of each symptom and its associated level of interference was used to determine the symptom's impact, as a basis for deciding whether the consultation behaviour was incongruous or not. An alternative approach could have been use of information based on GP opinion of what constitutes minor and serious symptoms. However, as consultation behaviour is heavily influenced by the perception of the person who has the symptom, it seemed appropriate to use the respondents' own assessments of their symptoms. If the consultation behaviour could not be explained by the respondents' judgements about a symptom's level of severity or interference with their daily lives (frequently identified as two of the key drivers of help-seeking behaviour in the literature) it seemed reasonable to call such behaviour incongruous. This approach avoided making external judgements about the appropriateness of the consultation behaviour. The fact that many symptoms rated by GPs as low seriousness were rated as high impact by patients and symptoms rated by GPs as high seriousness were rated as low impact by patients highlights the importance in differences in perceptions and the importance of considering the patients perspective when examining whether consultation behaviour is incongruous.

It is likely that some of the incongruous consultation behaviour identified in this study was appropriate. Low impact consultations for symptoms that the respondent thought indicated a potentially serious condition which warranted medical attention regardless of its level of severity or interference with daily activities probably constitutes appropriate behaviour. In our study, symptoms rated by a sample of GPs as more likely to be indicative of a potentially serious condition (level 4 and 5 symptoms) had a higher proportion of low impact consultations than minor self-limiting (level 1 and 2) symptoms. However, only 14% of the 254 low impact consultations occurred for potentially serious symptoms, while nearly half (45%) of all low impact consultations occurred for common minor self-limiting symptoms (reflecting potentially inappropriate behaviour).

Similarly, some of the high impact non-consultations reported in this study may have been appropriate behaviour. For example, high impact symptoms associated with a chronic condition that was already under GP management may have been less likely to result in a GP consultation in the previous two weeks. Although we know that about 65% of the high impact non-consultations identified in this study were made by people with a chronic condition we do not know if the symptom being reported was associated with this chronic condition or entirely independent of it. As a result we cannot identify how many of the high impact non-consultations in this study are related to a chronic condition.

In addition, as this was a cross-sectional study which only examined symptom experience and management actions in the previous two weeks we do not know if people had previously consulted their GP about their symptom(s). In order to explore this further we examined the proportion of symptoms resulting in a high impact non-consultation for which prescription medicine had been taken in the last two weeks. While not all symptoms are managed by prescription medicines, this data provides an approximate indication of what proportion of the symptoms associated with a high impact non-consultation were related to problems already under GP management. We found that only 18% of the 1384 symptoms associated with high impact non-consultations were being managed with prescription medication. This suggests that while some of the high impact non-consultations reported in this study may not have been inappropriate many possibly were.

This paper examined GP consultations only, and not consultations with other primary care health professionals. The development in the UK of primary care teams (including practice nurses and community pharmacists) has increased the range of healthcare professionals available for advice. UK government policy has advocated greater use of these individuals for the management of common symptoms and self-limiting illness [17]. As a result it may have been inappropriate to consider consultations with the wider primary care team for low impact symptoms as incongruous consultation behaviour.

The study response rate was low, an increasingly common problem in epidemiological research [18-20]. Despite the low response rate, the relatively large sample size and recruitment of practices from a wide variety of geographical and socio-economic areas ensured that most demographic and socio-economic groups (with the exception of non-whites) were well represented [13]. This allowed important sub-group analysis and provided a good level of generalisability for people of working age living in the UK. The dataset used in this paper has already been shown to produce symptom prevalence rates comparable with other studies [13] and similar proportions of service use as other studies [4], suggesting low response bias, although, our results should be interpreted with caution.

We adjusted for a wide range of demographic, socio-economic, health and symptom management factors. However, other potentially important characteristics may not have been measured (e.g. patient concern about symptoms, GP characteristics). Some residual confounding may have occurred as a result. Although efforts were made to minimise recall bias by asking about symptoms experienced in the previous two weeks, some bias may still have occurred. Finally, small numbers in each of the incongruous consultation behaviour groups for each of the individual symptoms meant that we could not examine whether characteristics associated with incongruous consultation behaviour differed for specific symptoms.

### Comparison with existing literature

While there is a reasonably large literature on why people consult [3], studies which do not control for symptom severity and interference are limited in the information they provide. Few studies have sought to quantify incongruous consultation behaviour or examine which factors are associated with being a low impact consulter or a high impact non-consulter. The most directly comparable study was undertaken by Hannay and Maddox in 1975 [21]. They examined what proportion of people in their UK study had symptoms that were part of the 'medical iceberg' (symptoms without a health professional consultation but which the patients rated as being serious, causing severe pain or causing severe disability) and what proportion had symptoms that were part of the 'medical trivia' (symptoms referred to a health professional which they did not consider serious and for which there was no pain or disability). Hannay and Maddox found that among participants with at least one symptom in the previous two weeks, 10.7% had symptoms that were part of the medical trivia and 25.9% had symptoms that were part of the medical iceberg; figures not dissimilar to our 8.3% and 37.1% respectively. Like us they also found little overlap between the two incongruous consultation behaviour groups: 6.9% were part of both the 'medical iceberg' and the 'medical trivia', compared with the 2.2% identified as low impact consulters and high impact non-consulters in our study. This small overlap suggests that while consultation behaviour in any one individual can vary depending on the symptoms involved, most individuals have discrete patterns of consultation behaviour. Like us, Hannay found a wide range of demographic, socio-economic and health variables were associated with being part of the 'medical iceberg' [22]. Unlike us, he also found a wide range of demographic and socio-economic variables were associated with being part of the 'medical trivia'. More consistent with our findings, he also reported that participants with poorer health were more likely to be part of the 'medical trivia'.

In a later study, Ingham and Miller reported that the most discriminating factor between those consulting and not consulting a GP for mild symptoms was the patient's own assessment of the likely cause of the problem [6]. People attributing a physical cause to their symptom were much more likely to consult than those attributing a psychological cause. In our study there were no clear differences in the proportion of physical and psychological symptoms resulting in low impact consultations.

Other studies have examined incongruous consultation behaviour for individual symptoms or conditions. Lydeard and Jones compared those consulting for dyspepsia with those not consulting for dyspepsia [23]. They found that 21% of non-consulting patients reported severe symptoms and 30% had symptoms more than 3 times a week, while 20% of consulters had mild symptoms and 27% experienced their symptoms less than once a week. Further examination of these two incongruous groups found no significant differences between them in how long they had suffered with the symptom, their knowledge about the symptom or the extent of their lay networks. There was however a significant difference between the groups in their beliefs and concerns about indigestion. Nearly three-quarters (74%) of the consulters were concerned that indigestion could lead to a serious or fatal condition, compared with 17% of the non-consulters. In particular, consulters were significantly more concerned about cancer and heart disease than the non-consulters. Concern about the cause of the symptom has also been reported as one of the main explanations for people consulting about other low impact symptoms, including cough [24] and irritable bowel symptoms [25]. We did not collect data on the participant's levels of concern about their symptoms. However, the fact that "*Feeling that I need reassurance or advice from a health professional*" was one of the factors found to be independently associated with being a low impact consulter supports the idea that people's perceptions about the seriousness of the symptom and concern about its underlying cause are important when deciding whether or not to consult a GP, regardless of its severity or interference on daily activities.

Lack of concern or failure to recognise a symptom as potentially indicative of ill health is a commonly reported explanation for people not consulting about symptoms associated with serious disease [26-29]. Corner et al. found that symptoms of lung cancer, even when severe and disruptive, are often interpreted as normal and not recognised as being serious or warranting medical attention [26]. Studies of urinary symptoms have consistently found that only about half of those with severe or bothersome urinary symptoms consult a doctor [27,28] with lack of awareness [29] or lack of concern [28,29] about symptoms the key reasons for failure to consult with these symptoms. Recent qualitative research has suggested several other reasons to account for people not consulting about high impact symptoms including fear or denial about the seriousness of the symptoms, barriers to accessing healthcare services, a sense of being unworthy of medical care, a perceived lack of effectiveness of healthcare services and medicines, the need to prioritise health problems and the need for independence [26,30-33]. It is not possible to know how often each of these reasons accounted for non-consultation in our study. However, our findings do support the suggestion that people sometimes feel undeserving of treatment with "*Feeling that I don't want to waste the GPs time*" independently associated with being a high impact non-consulter and "*Feeling that a symptom would not be viewed as important by others" *being significantly associated with being a high impact non-consulter after adjustment for age and sex. We also found evidence to support a perceived lack of effectiveness of healthcare services and medicines. "*My previous experience of healthcare services and medicines" *and *"Others previous experience of healthcare services and medicines" *were found to be important to high impact non-consulters. The fact that these two statements were significantly associated with being a high impact non-consulter after adjustment for age and sex suggests that people may not have consulted about their high impact symptoms because of previous bad experiences with particular services or because treatments had not worked for them in the past. It is important to understand why some people do not consult for symptoms of high impact, since many may benefit from diagnosis, treatment or advice.

## Conclusions and implications for future research

This was one of the first studies to examine incongruous consultation behaviour for a range of symptoms. It showed that around a fifth of all symptoms occurring in the community resulted in incongruous consultation behaviour based on respondents' own rating of their symptoms' impact. Most of the incongruous consultation behaviours related to high impact non-consultation. While a proportion of these high impact non-consultations were likely to be for symptoms associated with a chronic condition that was already known about and being managed, our findings suggest that many of these high impact non-consultations were associated with symptoms that were not known about or being treated. More research is needed to examine incongruous consultation behaviour and the potential impact that it has on both the public's health and health service use. In particular, future studies should further explore patients' beliefs and anxieties about the meaning of their symptoms as well as differences in attitudes towards seeking health care.

## Ethical approval

Ethical Approval for the study was granted by the Fife and Forth Valley Research Ethics Committee as part of the NHS Multi-Centre Research Ethics Committee for Scotland (Ref. 06/S0501/71).

## Competing interests

The authors declare that they have no competing interests.

## Authors' contributions

AME and PCH had the original idea for the research and developed the proposal. AM and AME conducted the data collection and analysis. AME wrote the first and subsequent drafts of the paper. All authors contributed to the scientific development of the paper, commented on successive drafts and agreed to the final manuscript.

## Funding

The study was funded by a Wellcome Trust Research Career Development Fellowship for Dr Alison M Elliott (grant reference number: 078176/Z/05/Z). The funders had no role in study design, in the analysis and interpretation of data, in the writing of the report, or in the decision to submit the article for publication.

## Pre-publication history

The pre-publication history for this paper can be accessed here:

http://www.biomedcentral.com/1471-2296/13/21/prepub
